# The Sasang Constitution as an Independent Risk Factor for Metabolic Syndrome: Propensity Matching Analysis

**DOI:** 10.1155/2013/492941

**Published:** 2013-11-20

**Authors:** Eunsu Jang, Younghwa Baek, Kihyun Park, Siwoo Lee

**Affiliations:** Department of KM Health Technology Research Group, Korea Institute of Oriental Medicine, 1672 Yuseongdae-ro, Yuseong-gu, Daejeon 305811, Republic of Korea

## Abstract

The Sasang Constitutional Medicine is a traditional Korean customized medicine that classifies people into four types: Tae-eumin (TE), Soyangin (SY), Soeumin (SE), and Taeyangin. The aim of this study was to show whether the Sasang Constitution (SC) could be an independent risk factor for the metabolic syndrome (MS). Totally, 3,334 subjects from 24 Korean medicine clinics participated in this study. A one-way ANOVA for the continuous variables and a chi-square test for the prevalence of MS were conducted. A logistic regression was conducted to calculate the propensity score and the odds ratios (ORs). The prevalence for MS in TE, SY, and SE was 50.6%, 30.9%, and 17.7% (*P* < 0.001) before matching, and 36.7%, 28.6% and 28.2% (*P* = 0.042) after matching, respectively. The TE was associated with an increased OR for MS compared with the SE and SY in both crude (OR 4.773, 95% CI 3.889–5.859, and OR 2.292, 95% CI 1.942–2.704, resp.) and matched groups (OR 1.476, 95% CI 1.043–2.089, and OR 1.452, 95% CI 1.026–2.053, resp.). This study reveals that the SC, especially the TE type, could be considered as a risk element for MS even in people with otherwise similar physical characteristics.

## 1. Introduction

Metabolic syndrome (MS) has a high prevalence worldwide and has important implications for the health care sector [1, 2]. MS, known as cardiometabolic syndrome or syndrome X [3], is defined as a combination of at least three of following five medical disorders: hypertension, hyperglycemia, elevated triglycerides, low high-density lipoprotein cholesterol, and abdominal obesity [4]. It has been suggested that MS plays a pivotal role in the occurrence of cardiovascular disease and the subsequent development of type 2 diabetes mellitus (DM) [5, 6]. Therefore, it is very important to clearly understand this role, to perform risk assessment for MS, and to detect prior risk factors [7]. Several studies have revealed that acquired risk factors, such as body mass index (BMI), aging, physical inactivity, hormonal imbalance, and stress, as well as inborn factors are strongly associated with MS [8–10]. Among them, innate factors have been continuously demonstrated as important risk factors for MS and a lot of studies have controlled for physical characteristics including age and weight to find inborn risks or refine some congenital factors clearly [9, 11, 12].

Many statistical methods are used in clinical research to reduce the selection bias for different physical characteristics. Recently, matching with the propensity score has been widely used [13]. Propensity matching is a type of statistical method consisting of the experimental and comparative groups with homogenous characteristics and it is relatively more correct to compare the effects of an intervention. 

Sasang Constitutional Medicine (SCM) is a traditional Korean customized medicine that classifies people into four types: Tae-eumin (TE), Soyangin (SY), Soeumin (SE), and Taeyangin (TY) [14]. The general constitutional traits including psychological and physiological symptoms are known to be clinically and congenitally different from one type to another [15]. Many studies scientifically revealed that specific SC types were prone to specific chronic diseases including hypertension, DM, and abdominal obesity. This propensity matching could be managed as an innate risk factor, following the SCM theory [16–20]. A recent study for MS revealed a significant association with the Sasang Constitution (SC) and suggested that the TE type has a higher prevalence and odds ratio (OR) for MS compared with the SE type [19]. However, it is difficult for this study to show the pure effect of SC types on MS because the general characteristics including age and BMI in that study were significantly different from one constitution to another. We hypothesized that the SC type could be also associated with MS even when controlling for similar physical characteristics. This study was aimed to show whether the SC type is an independent risk factor for MS by using propensity matching.

## 2. Materials and Methods

### 2.1. Subjects

**Figure 1:**
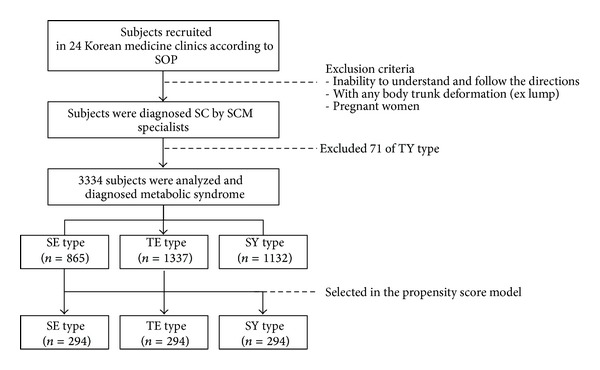
The study flow chart. SOP: Standard Operation Procedure, SCM: Sasang Constitutional Medicine, SC: Sasang Constitution, TY: Taeyangin, SE: Soeumin, TE: Tae-eumin, and SY: Soyangin.

This cross-sectional study was conducted from Nov 2006 to Jul 2012. All the clinical data including the MS components and individual SC types were derived from the Korean Constitutional Multicenter Bank in the Korea Institute of Oriental Medicine (KIOM), which is the biggest clinical database of SC in Korea. Subjects from 24 Korean medicine clinics participated in this study. Individuals who were physically unable to follow the researcher's instructions and those with deformation in the measurement locations were excluded. Pregnant women were also excluded. The 71 TY types were excepted from analysis due to their rarity in the Korean population. A total of 3,334 subjects over the 20s were analyzed in this study (Figure 1). The eligibility criteria were previously described in detail [20]. This study was approved by the Institutional Review Board in KIOM (I-0910/02-001). All participants agreed to join this study, and written informed consent for participation was obtained from every subject.

### 2.2. Data Collection

The blood pressure was measured at rest in the left upper arm. Body measurement data including waist circumference were collected from each subject after the upper clothing was taken off. Blood samples were obtained from the left brachial vein after more than 12 hours of fasting. Triglyceride (TG), high-density lipoprotein (HDL) cholesterol, and fasting blood glucose levels were tested after transferring all the blood samples from the 24 individual clinics to a single authorized institution. All process for the data acquisition were conducted according to a standard operation procedure that was documented for the Korea Constitution Multicenter Study [21].

### 2.3. Sasang Constitutional Diagnosis

For accurate SC diagnosis, firstly this study warranted the qualification of the experts of SCM. Those experts who had more than 5 years of experience in clinical practice of SCM had been collected from national wide hospitals. They diagnosed the individuals' SC type by examining his/her appearance, voice, temperament, body shape, and physiological symptoms in each hospital. Secondly, the medical chart review for the participants who had taken SC-specific pharmaceuticals more than one month following experts SC diagnosis was conducted as an additional way to identify the participant SC type because it is known that the inappropriate prescription caused adverse herbal reactions [22]. Only those, who had shown good improvements in their chief complaints as well as in physiopathological symptom became the candidates of this study. Lastly, the experts of SCM with clinically sufficient qualification reconfirmed the SC type of the participants. The similar procedures for SC diagnosis were described at a former study [21].

### 2.4. Diagnostic Criteria of MS

In this study, the National Cholesterol Education Program Adult Treatment Panel III (NCEP ATP III) guidelines applied to the definition of MS, which required the presence of at least 3 out of 5 factors [4]. Some criteria were modified as follows. DM was diagnosed with a fasting plasma glucose ≥100 mg/dL or taking medicine for the treatment of DM, following the preventive medical trend according to the International Diabetes Federation (IDF) criteria [23]. Hypertension was diagnosed with a diastolic blood pressure (DBP) ≥90 mmHg, a systolic blood pressure (SBP) ≥140 mmHg, or the use of medicine for the treatment of high blood pressure following the guidelines of the 7th Report of the Joint National Committee (JNC) [24]. Abdominal obesity was diagnosed with a waist circumference (WC) ≥90 cm for males and ≥80 cm for females [25]. Hypertriglyceridemia was diagnosed with TG ≥ 150 mg/dL. Low HDL cholesterol was diagnosed with HDL cholesterol <40 mg/dL in males and <50 mg/dL in females [4]. 

### 2.5. Propensity Score Matching

**Figure 2:**
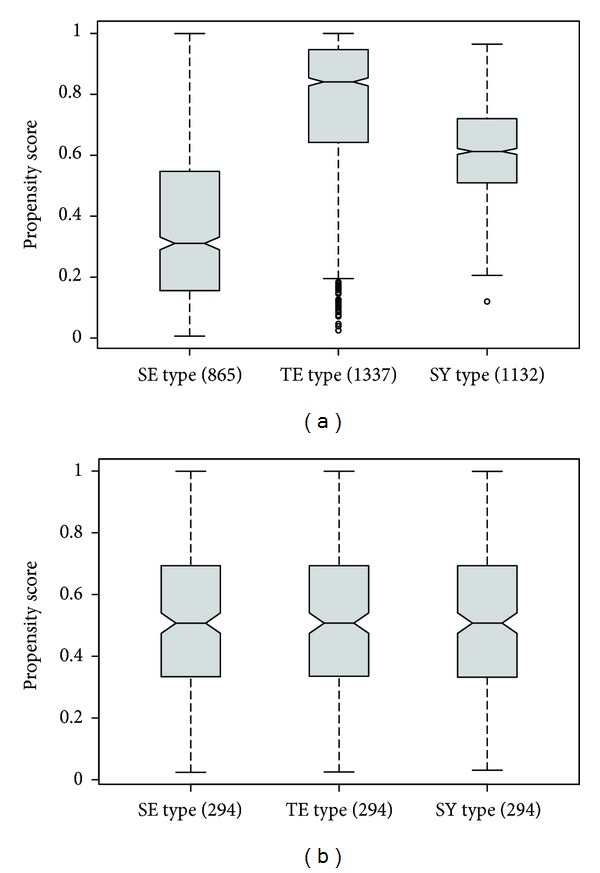
Comparison of the propensity score among SC types before and after propensity matching: (a) propensity score before matching, (b) propensity score after matching. SE: Soeumin, TE: Tae-eumin, and SY: Soyangin.

The physical characteristics were matched using a propensity score consisting of age, height, weight, and BMI. We calculated the propensity score (PS) for each SC type using a logistic regression in males and females separately. A matching process was conducted with a minimum distance scoring method, and each PS of the SY and TE type was matched with the closest PS of the SE type.  Figure 2 shows the change in PS distribution among the matched SC types. 

### 2.6. Statistical Analysis

A one-way ANOVA was conducted to compare the general and clinical variables (Scheffé's post hoc analysis), and a chi-square test was used to compare the prevalence of MS according to the SC before and after propensity matching. To evaluate whether the SC is an independent risk factor for MS, a logistic regression was conducted to calculate the odds ratios (ORs) for MS in the propensity matching group as well as in the crude. The likelihood ratio test (LRT) was calculated to clarify how well the crude and chosen model fit the data. The *P* value of the LRT was <0.001 (chi-square value: 272.474) in crude and 0.044 (chi-square value: 6.266) in the propensity score model. We performed all analyses using SPSS 21.0 software (SPSS Inc., Chicago, IL, USA), and *P* values <0.05 were regarded as statistically significant. 

## 3. Results

The number of the SE, TE, and SY types was all 294 after propensity matching, from 865, 1337, and 1132 in crude, respectively. The general characteristics were significantly different among the SC types before matching (*P* < 0.001), but no statistically significant differences were shown after matching. The details were shown in Table 1. 

The examination numerical values of 5 components for MS were significantly different among the SC types before matching (*P* < 0.001). After matching, there were significantly different in those values of SBP (*P* = 0.014), TG (*P* = 0.035), HDL (*P* = 0.028), and WC (*P* = 0.02).  Table 2 shows the clinical details of the 5 components of MS.

**Figure 3:**
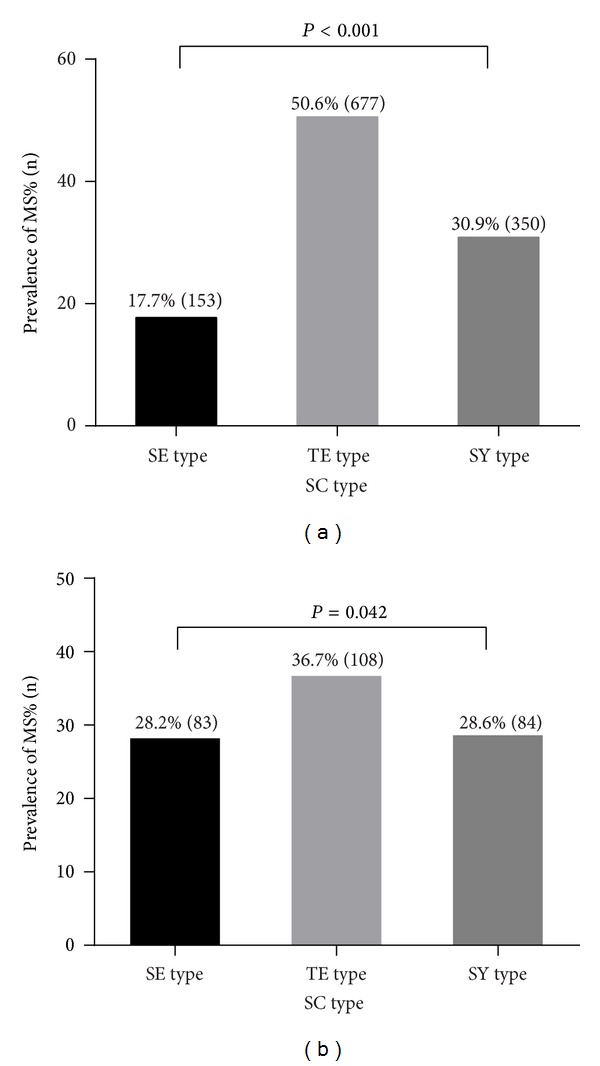
The prevalence of MS according to the SC type before and after propensity matching. (a) prevalence of MS before matching, (b) prevalence of MS after matching. MS: metabolic syndrome, SC: Sasang Constitution, SE: Soeumin, TE: Tae-eumin, and SY: Soyangin.

Before matching, the prevalence for MS was significantly different as 50.6% in the TE type, 30.9% in the SY type, and 17.7% in the SE type (*P* < 0.001). The prevalence for MS was still different as 36.7% in the TE type, 28.6% in the SY type, and 28.2% in the SE type, respectively (*P* = 0.042). This result suggested that the TE type had a relatively higher prevalence for MS than other types despite similar physical conditions among the groups (Figure 3).

Table 3 showed the ORs for MS before and after matching between the SC types. The result revealed the TE type was associated with an increased prevalence of MS compared with the SE type in both crude (OR 4.773, 95% CI 3.889–5.859, *P* < 0.001) and propensity matched groups (OR 1.476, 95% CI 1.043–2.089, *P* = 0.028), respectively. The TE type was associated with increased OR for MS compared with SY type in crude (OR 2.292, 95% CI 1.942–2.704, *P* < 0.001), and OR for MS in TE type still remained associated with increased OR (OR 1.452, 95% CI 1.026–2.053, *P* = 0.035) after matched group. Lastly, SY type was associated with increased OR for MS compared with SE type in crude (OR 2.083, 95% CI 1.679–2.583, *P* < 0.001), whereas OR of SY type was not different compared with the SE type (OR 1.017, 95% CI 0.711–1.455, *P* = 0.927) after matched group. This finding revealed the TE type may be an important independent risk factor for MS regardless of the age and BMI.

## 4. Discussion

The aim of this study is to show whether the SC type is an independent risk factor for MS despite homogenous patient characteristics. Summarizing this study, the prevalence for MS was significantly different according to SC types in both before and after matched groups. The TE type had significantly increased ORs for MS compared to the SE and SY types in crude (OR 4.773, 95% CI 3.889–5.859, *P* < 0.001 and OR 2.292, 95% CI 1.942–2.704, *P* < 0.001, resp.) and matched groups (OR 1.476, 95% CI 1.043–2.089, *P* = 0.028 and OR 1.452, 95% CI 1.026–2.053, *P* = 0.035, resp.). This result implies that the SC type can affect the prevalence of MS and can act as an independent risk factor for MS, despite similar body figure and age.

In detail, the general characteristics of the enrolled subjects were significantly different according to SC types; thus, it was very difficult to directly compare the primary outcome among SC types. Therefore, the SY, SE, and TE types were matched through the propensity score. After matching, general characteristics including age and BMI showed no significant difference among SC types. This meant that there was minimal selection bias among matched SC groups and all SC types become almost homogenous.

This study revealed that all of the clinical examination numerical values of MS were similar to previous individual studies before matching [16, 17, 20] and those values of individual SBP, TG, and WC were significantly different according to SC types after matching. Further, this study newly added that the HDL cholesterol test values of the TE significantly differed from the SE type, which had been suggested in a previous genetic study demonstrating that serum lipids are associated with specific SC type [21].

There was difference in MS prevalence according to SC types and the TE type had a higher OR for MS compared to each the SE, and SY types in both before and after matched groups. It meant that it could apply not only to the SC types with different characteristics but also even to the SC types with similar characteristics. 

This result derived from a matching analysis using the propensity score of age and BMI, which is a good method to reduce selection bias, advanced the previous study [19]. Therefore, this is a stronger indication that the SC types especially TE type could be an independent risk factor for MS than the previous study because age and BMI were not controlled in there. 

This study also strongly supported the original hypothesis in SCM that the disease could occur differently from one constitution to another, by demonstrating that SC type was an independent risk factor for MS and the TE type is prone to developing MS despite similar biological characteristics. 

We think that our findings showed the presence of SC and enhanced evidence level of SC as an independent risk factor for MS.

There are some weaknesses in this research. The exact sensitivity and specificity of SC diagnosis by the experts has not been known yet, even though a local pilot study insisted that the consistency of the assignment among different specialists with high level of qualification in SCM showed substantial agreement over 0.7 Kappa coefficient [26]. The consistency of the assignment of the experts in this study supported by herbal response for participants might be higher than that of former study. However, there was no empirical data to support the high consistency of assignment among the experts in this study. In future work, it is highly necessary to reveal the real concordance of the assignment and the exact sensitivity and specificity among different experts. 

Actually, there are many types of traditional customized theory existed in Asian countries: Ayurvedic medicine in India, SCM in Korea, Chinese Constitutional Medicine in China, and Kampo Ikkando medicine in Japan. These traditional customized medicines all have common traits that they classify people into their own categories and practice intervention to the patients accordingly. The classifying or diagnostic methods are completely dependent on the experts' opinions which are mostly subjective. So many researchers have tried to make an objective diagnostic tool, but when it comes to diagnostic accuracy, it is quite controversial among researchers as some suggested positive result [27], while some negative ones [28, 29]. There is no established and prevailing diagnostic tool at this moment. That was why we adopted the experts' opinion with the medical records of patients who had no adverse effect for SC-specific pharmaceuticals as a clinical proof of constitution confirmation. 

Furthermore, this study did not show a direct causal relationship between SC and MS because of our cross-sectional study design. We did not regulate for acquired underlying risk factors in MS, such as exercise habits or diet. Further study considering environmental factors and/or a longitudinal design are also necessary.

## 5. Conclusions

This study reveals that SC types, especially TE type, could be an independently important risk factor for MS. Therefore, this result suggests that SC types should be considered as a risk element for MS even for people with similar physical characteristics.

## Figures and Tables

**Table 1:** General characteristics according to the SC before and after propensity matching.

Variables	Before matching				After matching			
	SE type	TE type	SY type	*P*	SE type	TE type	SY type	*P*
Numbers	865	1337	1132		294	294	294	
Height (cm)	161.7 ± 8.1	162.5 ± 8.6	161.2 ± 8.4	<0.001^c^	161.9 ± 8.3	162 ± 8.3	160.8 ± 8.9	0.185
Weight (kg)	55.9 ± 8.6	67.5 ± 11.1	59.7 ± 9.5	<0.001^a,b,c^	59.9 ± 8.9	60 ± 8.7	59.2 ± 9	0.448
BMI (kg/m^2^)	21.3 ± 2.5	25.5 ± 3.1	22.9 ± 2.7	<0.001^a,b,c^	22.8 ± 2.4	22.8 ± 2.5	22.8 ± 2.5	0.989
Age (yrs)	46.6 ± 14.7	51.3 ± 14.7	49.3 ± 14	<0.001^a,b,c^	49.8 ± 13.4	49.1 ± 16.1	49.6 ± 14	0.848

Data shown are the mean ± SD or numbers. ^a^Soeumin and Soyangin differ significantly, ^b^Soeumin and Tae-eumin differ significantly, and ^c^Soyangin and Tae-eumin differ significantly.

SC: Sasang Constitution; TE: Tae-eumin; SE: Soeumin; SY: Soyangin; BMI: body mass index.

**Table 2:** Clinical figure of five components for MS according to SC before and after propensity matching.

Variables	Before matching				After matching			
	SE type	TE type	SY type	*P*	SE type	TE type	SY type	*P*
SBP (mmHg)	116.4 ± 14.9	123.4 ± 15.8	118.4 ± 15.1	<0.001^a,b,c^	117.8 ± 14.2	120.7 ± 14.9	117.7 ± 13.8	0.014^b,c^
DBP (mmHg)	74.8 ± 11	79.1 ± 11	76.4 ± 11.1	<0.001^a,b,c^	76.1 ± 10.5	76.6 ± 10.9	76.3 ± 10.5	0.838
FBS (mg/dL)	94.7 ± 21.4	101.9 ± 30	99.6 ± 32.6	<0.001^a,b^	95.9 ± 21.1	96.6 ± 28.5	99.6 ± 36.1	0.259
TG (mg/dL)	102.8 ± 58.3	143.7 ± 91.6	126.5 ± 82	<0.001^a,b,c^	116.6 ± 70.7	131.7 ± 87.1	118.9 ± 69.8	0.035
HDL (mg/dL)	50.6 ± 12.7	44.5 ± 11.6	47.5 ± 12.4	<0.001^a,b,c^	48.9 ± 12.9	46.2 ± 11.7	47.3 ± 11.4	0.028^b^
WC (cm)	78.7 ± 8.2	89.8 ± 8.9	82.4 ± 8.4	<0.001^a,b,c^	82.2 ± 7.3	83.6 ± 8.5	81.9 ± 7.6	0.02^c^

Data shown are the mean ± SD. ^a^Soeumin and Soyangin differ significantly, ^b^Soeumin and Tae-eumin differ significantly, and ^c^Soyangin and Tae-eumin differ significantly.

SC: Sasang Constitution, SBP: systolic blood pressure, DBP: diastolic blood pressure, FBS: fasting blood sugar, TG: triglycerides, HDL: high-density lipoprotein cholesterol, WC: waist circumference, SE: Soeumin, TE: Tae-eumin, and SY: Soyangin.

**Table 3:** The odds ratios and 95% CI for MS before and after propensity matching according to the SC.

	OR (95% CI) for MS	
	Crude	After matching
SE type : TE type	1 : 4.773 (3.889–5.859)	1 : 1.476 (1.043–2.089)
	*P* < 0.001	*P* = 0.028
SY type : TE type	1 : 2.292 (1.942–2.704)	1 : 1.452 (1.026–2.053)
	*P* < 0.001	*P* = 0.035
SE type : SY type	1 : 2.083 (1.679–2.583)	1 : 1.017 (0.711–1.455)
	*P* < 0.001	*P* = 0.927

SC: Sasang Constitution, SE: Soeumin, TE: Tae-eumin, SY: Soyangin, MS: metabolic syndrome, OR: odds ratio, and CI: confidence interval.
